# What Makes Green Cities Unique? Examining the Economic and Political Characteristics of the Grey-to-Green Continuum

**DOI:** 10.3390/land3010131

**Published:** 2014-01-24

**Authors:** Daniel Miller Runfola, Sara Hughes

**Affiliations:** 1 National Center for Atmospheric Research, NCAR-FL2, 3450 Mitchell Lane, Boulder, CO 80301, USA; 2 Institute of Behavioral Science, University of Colorado at Boulder, Boulder, CO 80305, USA

**Keywords:** governance, policy, vegetative land cover, lawns, trees, parks

## Abstract

In the United States, urbanization processes have resulted in a large variety—or “continuum”—of urban landscapes. One entry point for understanding the variety of landscape characteristics associated with different forms of urbanization is through a characterization of vegetative (green) land covers. Green land covers—*i.e.*, lawns, parks, forests—have been shown to have a variety of both positive and negative impacts on human and environmental outcomes—ranging from increasing property values, to mitigating urban heat islands, to increasing water use for outdoor watering purposes. While considerable research has examined the variation of vegetation distribution within cities and related social and economic drivers, we know very little about whether or how the economic characteristics and policy priorities of green cities differ from those of “grey” cities—those with little green land cover. To address this gap, this paper seeks to answer the question *how do the economic characteristics and policy priorities of green and grey cities differ in the United States?* To answer this question, MODIS data from 2001 to 2006 are used to characterize 373 US cities in terms of their vegetative greenness. Information from the International City/County Management Association's (ICMA) 2010 Local Government Sustainability Survey and 2009 Economic Development Survey are used to identify key governance strategies and policies that may differentiate green from grey cities. Two approaches for data analysis—ANOVA and decision tree analysis—are used to identify the most important characteristics for separating each category of city. The results indicate that grey cities tend to place a high priority on economic initiatives, while green cities place an emphasis on social justice, land conservation, and quality of life initiatives.

## 1. Introduction

Urban development over the last four decades has resulted in a wide range of altered landscapes, from dense business districts to sparsely populated, residential suburbs [1–6]. Vegetative land cover is frequently altered by urbanization, but to varying degrees in different cities [7]. The political and economic decisions city governments make regarding the costs (*i.e.*, production, irrigation, maintenance [7–10]) and benefits (*i.e.*, human health, biodiversity [11,12]) of vegetative land cover result in an urbanization continuum that reflects the variety of approaches to urban development [13–16]. This vegetated continuum of urbanization has important implications not only because it is linked to land use and cover types, but also because it may reflect different governance approaches and economic priorities. These relationships are critical for research agendas ranging from household-scale human-environmental interactions [17–19] to regional impacts of global climate change [6,20–25].

Variation in vegetative land cover has been shown to be associated with a wide range of human and environmental outcomes, from the extent of urban heat islands [26], to social ties between neighbors [27], to changing water quality, soil profiles, runoff, and water biochemistry [28,29]. However, we do not have an equally rigorous understanding of how the economic characteristics and policy priorities of cities produce green or grey urban landscapes. To begin to fill this gap, this paper examines the variation of vegetation in urban environments in order to answer the research question: how do the economic characteristics and governance priorities of green and grey cities differ in the United States?

### 1.1. Why Urban Vegetation Matters

Understanding the mechanisms that result in vegetated landscapes is important, as vegetative greenness in cities has been shown to influence valued environmental and human outcomes both positively and negatively. For example, accessible green space—including land cover types such as lawns—has been shown to improve the formation of strong social ties among neighbors [27,30], reduce crime [31], decrease senior citizen mortality [32], increase cognitive development in children [33], reduce stress [34], and lead to a variety of other positive health outcomes [12,35]. Impacts of intensive lawn maintenance on biogeochemical cycling may also be large enough to merit inclusion in local or regional atmospheric dynamics: the annual vegetative growth associated with US lawns may be responsible for up to 17 Tg/y of carbon removal from the atmosphere [36,37].

However, increasing vegetative cover in urban areas comes with political and economic challenges for program management and maintenance [38]. A significant body of research has shown that lawn maintenance is an important driver of water use in urban areas [37,39–42]. Water quality may also be affected by increases in vegetative land covers through changes in nitrogen and phosphorus runoff from fertilizer application, which poses risks to the health of humans, plants and animals [29]. The need to finance and maintain trees and open space can also strain city budgets and may require new decision making processes [43].

### 1.2. City Governance and Vegetation

The variation in vegetative greenness within cities often corresponds to the racial and socio-economic characteristics of neighborhoods and other social units [44–48]. However, we know less about how and why vegetative greenness varies *between* cities. While the geographic location of a city clearly influences the availability of water and other inputs necessary to foster plant growth, the highly modified nature of urban environments means that geography is unlikely to entirely explain the variation in vegetation between cities (*c.f.* [37,49,50]). In particular, “green” and “grey” cities may have different economic characteristics and policy priorities that support decisions that prioritize vegetative greenness.

First, green cities may have different priorities, expressed through general policy initiatives, than grey cities. For example, highly vegetated cities may be more likely to prioritize environmental outcomes, quality of life goals, social justice, green space and green infrastructure; grey cities may be more likely to prioritize economic growth and development (though policy tradeoffs may complicate these relationships) (*c.f.* [51,52]). Second, green cities may be more likely to have specific land use policies that encourage greenness through densification, green building practices, brownfield redevelopment, and land conservation [53,54]. Third, green and grey cities may have different economic bases that drive and reflect policy priorities. For example, cities with a tourism-based economy are more likely to work with environmental organizations (and therefore more likely to be green) [55]. Finally, the challenges to development a city faces (economic barriers) may also be related to vegetative greenness. Green land cover requires available land; cities that face a shortage of land, or see land availability as a barrier to development, may also be less green. Cities where low levels of political support and a poor quality of life are acting as barriers to economic development may also be less green because they lack the organization and amenities that are associated with greening programs [38,56]. In this paper, we will examine the relationship between these economic and policy characteristics and the greenness levels of cities.

## 2. Methods

### 2.1. Data and Study Area

**Figure 1:**
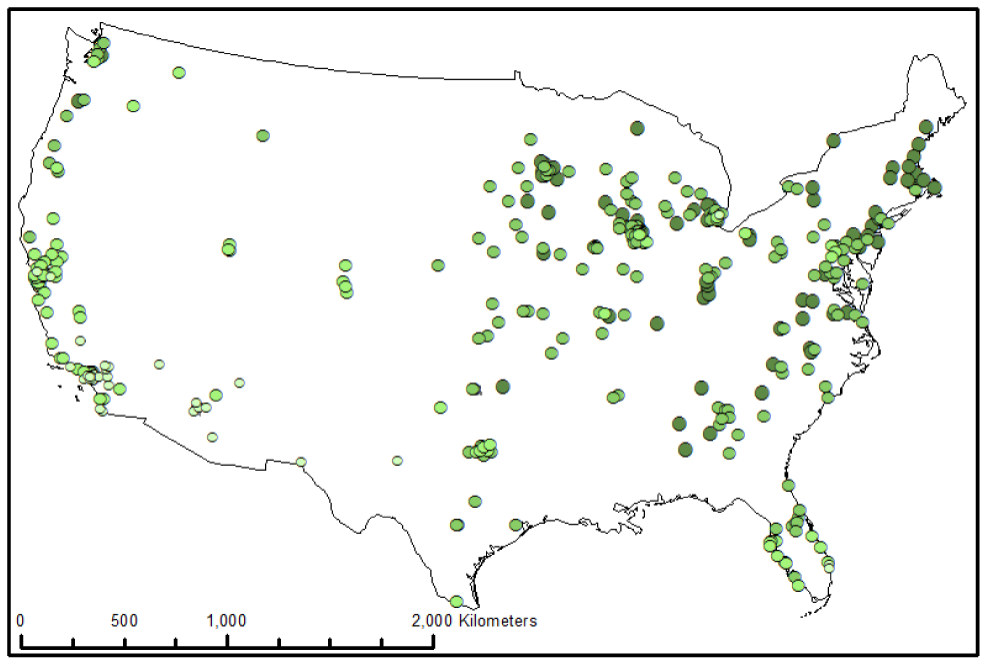
US Cities represented in the International City/County Management Association's (ICMA) 2010 Local Government Sustainability Survey and the 2009 Economic Development Survey. Darker green indicates higher vegetative greenness.

A total of 373 US cities are examined in this paper using two types of data: survey information and satellite observed vegetative greenness information (NDVI). These cities, a convenience sample based on responses to two International City/County Management Association's (ICMA) surveys, range in population size from 1,068 to 104,590 (2005 Census) and are located in nearly all 48 contiguous US states (see Figure 1).

In order to examine the political and economic characteristics of green and grey cities, responses to the ICMA's surveys on sustainability and economic development were used to characterize the cities along the four dimensions, or themes, described previously as potentially being related to the greenness of a city: (1) general policy initiatives; (2) land use/cover policies; (3) economic base; and (4) perceived economic barriers. The questions used for each sub-topic, as well as the variable name assigned to each question, are summarized in Table 1. The answer to each question was recorded as a binary value, for which an affirmative answer was given a 1 and a negative answer was given a 0.

This survey information is coupled with a metric of vegetative greenness for each city. Dozens of approaches to measuring vegetative greenness exist, each with its own strengths and weaknesses [37,50,57–65]. In this study, we utilize a single measurement of vegetative land cover—the Normalized Difference Vegetation Index (NDVI) measured using the MODIS satellite system [66]. While NDVI has been widely used to measure vegetation in a variety of urban settings [67–72], measurements taken with coarse-resolution tools such as MODIS are limited in a number of key ways. For example, research has indicated that these measurements are limited in their usefulness for approximating landscape characteristics—*i.e.*, fine-scale vegetation patch spatial structures—within urban environments (see Stefanov and Netzband [68] for a detailed description of the many limitations associated with using MODIS NDVI for intra-city analysis). To mitigate these concerns, we examine only inter-city comparisons of NDVI, using a coarse definition of “above” or “below” average vegetative density for each city. Results from comparing a subset of towns (N = 26) for which high resolution vegetation data are available suggest that our approach provides a relatively high degree of accuracy (77% agreement) while still allowing for a large-N analysis of the relationship between urban governance strategies and vegetative greenness. Future research should compare such findings across a broader range of biomes, vegetative metrics, and temporal scales to better inform future, broad-scope systematic studies of urban vegetation, as well as validate this study's findings.

To facilitate the NDVI classification, satellite information was retrieved from the Global Land Cover Facilities US Vegetation Index product, which is derived from daily MODIS 250 m resolution red and near infrared bands [59]. Composite images are produced every 16 days using data quality (*i.e.*, cloud coverage) and maximum vegetative index values across each time step. These images are then classified using a Normalized Difference Vegetation Index (NDVI). Values approaching 0 indicate very sparse vegetation, and dense vegetation is indicated by values approaching 1. To estimate the density of vegetation across each of our 373 cities, the average NDVI value from 2001 to 2006 was retrieved within the US Census Place geographic boundaries associated with each city. Cities with above-average NDVI values were categorized as “Green” cities, while below-average cities were categorized as “Grey”.

### 2.2. Analysis

**Figure 2:**
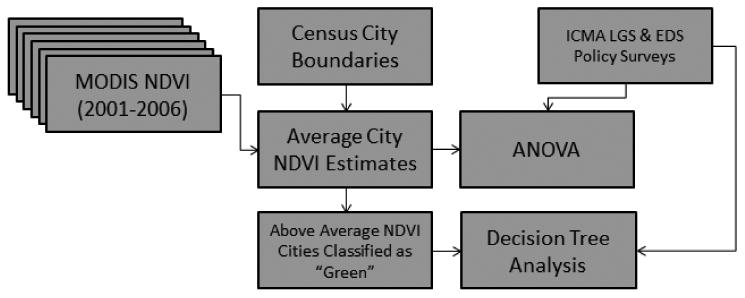
Technical flow chart of steps and data used in this analysis.

Each step of the data collection and analysis process is summarized in Figure 2. To examine whether general policy initiatives, land use/cover policies, economic base and economic barriers distinguish above- and below-average greenness cities, we implement two different procedures. First, an analysis of variance (ANOVA) is performed to explore which factors (summarized in Table 1) may be used to identify groups of cities with NDVI means that have statistically significant differences. We then perform a decision tree classification—a nonparametric technique which identifies the strongest variables for use in distinguishing multiple discrete classes of data (in our case, above- and below-average greenness cities). The decision tree model allows us to both test the robustness of our ANOVA results and develop a better understanding of the context(s) in which variables are more or less important in differentiating green and grey cities.

Decision tree classification techniques have become increasingly popular in remote sensing research for their ability to provide nonparametric “data mining” approaches to classifying satellite imagery [62]. In our case, the decision tree operates by first taking the full population of cities, and “classifying” them according to thresholds using ancillary data-for example, all cities which have a strong tourism economic base may be classified as “above average greenness”, which will result in some number of both correctly and incorrectly classified cities when compares to our observed NDVI-based estimates. This is performed iteratively, so further splits can be created amongst sub-groups of the data at every tier of the tree. Splits are recursively determined by minimizing the variance within each defined class (measured *via* the metric *G*^2^)—*i.e.*, the algorithm attempts to correctly classify the largest number of cities within each split. A lower *G*^2^ value indicates a better “fit” at a given node within the tree. Based on the position of a splitting variable within the tree the relative importance of variables in defining a cities category (below or above average vegetative greenness) can be identified.

## 3. Results

### 3.1. Measuring Vegetative Greenness, Economic Characteristics, and Policy Priorities

Descriptive results for the economic characteristics and policy priorities of the cities can be found in Table 2. To examine how representative our dataset—which includes cities that responded to both the sustainability and economic ICMA surveys—is of all cities that responded to either survey, we report the full sample information as well. While many cities (73%) have economic policy initiatives (compared to 67% in the full sample), far fewer have environmental (28%; 20% in the full sample) or social justice (11%; 9% in the full sample) policy initiatives. The most commonly occurring sustainability policy initiative of any kind is the installation of trails for hiking or biking (75%; 61% in the full sample). Many cities (29%; 28% in the full sample) in our sample had a manufacturing economic base, while only 9% (also 9% in the full sample) of cities had a telecommunications and technology economic base. Quality of life was the least frequently reported economic barrier (5%; 6% in the full sample) while land availability was most frequently reported as an economic barrier (48%; 50% in the full sample). Very few cities have policies supporting economic incentives to promote environmentally friendly development (8%; 3% in the full sample), and urban gardens are the most common sustainability oriented land use/cover policy (38%; 29% in the full sample). Only 21% of responding cities have policies for preserving open space (15% in the full sample), while 32% have both brownfield redevelopment and land conservation policies (22% in full sample for both).

**Figure 3:**
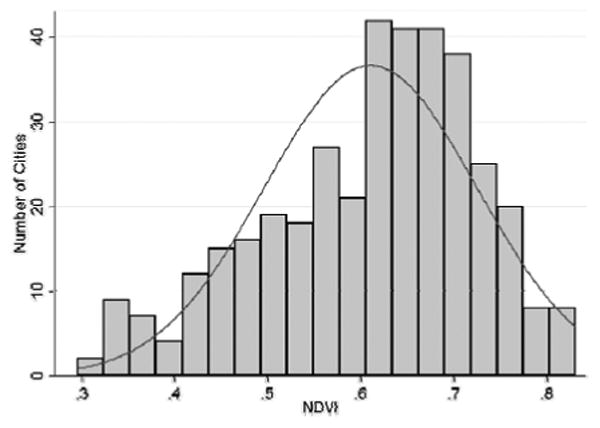
Distribution of c ities in terms of vegetative density. The solid line represents a normal density curve of the data. Higher values indicate dense vegetation, lower values indicate sparse vegetation.

Vegetative density as measured using MODIS using NDVI ranged from 0.296 to 0.832, with a mean of 0.61 (σ = 0.114). The distribution is slightly right-skewed (Figure 3). Further, there is an apparent spatial east-west trend of vegetation across the United States, with higher levels of vegetation generally being found to the east (Figure 1).

### 3.2. Comparing Grey and Green Cities

The results from the ANOVA can be seen in Table 3. Because each of our variables (listed in Table 2) are binary variables, this ANOVA is testing the null hypothesis that, for each variable, the mean NDVI value in cities is the same for cities that responded both positively and negatively to each question. Significant *F* statistics indicate a statistically significant difference between groups, and within-group means can be examined to assess the directionality associated with having a given policy or economic focus. In our analysis, four variables were significant in their relationship to vegetative greenness (alpha < 0.05): having a general economic (−) and/or social justice (+) governance focus, having land conservation policies in place (+), and reporting land as an economic barrier to development (+).

**Figure 4:**
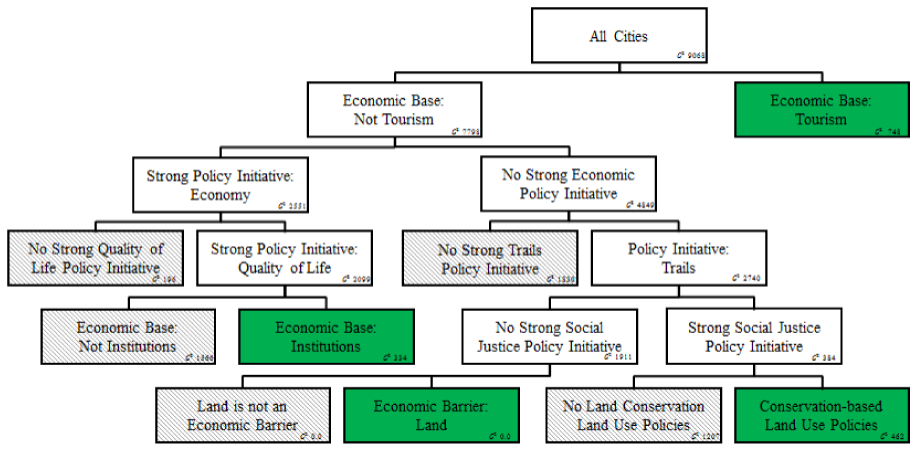
Decision Tree analysis results. Overall r^2^ = 0.3; AICc = 1,831.42; Number of splits = 8.

The results from the decision tree analysis can be seen in Figure 4. The decision tree shows that a tourism-based economy (+) has the greatest ability to explain variance in green land cover, but the relatively high *G*^*2*−^ value in the two nodes it splits into suggests that the variable does not have strong explanatory capabilities on its own. To improve model fit, cities that do not have a tourism-based economy are further differentiated according to whether or not they have strong economic policy initiatives. Cities with strong economic policy initiatives are differentiated by whether they also have strong quality of life policy initiatives, and those cities without quality of life policy initiatives have below average vegetation cover. Among cities that do have strong quality of life policy initiatives, those that have an institutional economic base (universities, military, *etc.*) have higher vegetation levels than average.

Cities without strong economic policy initiatives are differentiated first by whether or not they also have a strong trails policy initiative and those cities without have lower vegetation levels than average. Cities that do have strong trails policy initiatives are first differentiated by whether they also have strong social justice policy initiatives. Cities that do not have a strong social justice policy initiative and also identify land availability as a barrier to economic development have higher vegetation levels than average. Cities that do have strong social justice policy initiatives and also have conservation-based land use policies have higher vegetation levels than average.

In order to test both the robustness and importance of spatial patterns in the MODIS NDVI measurements, a second decision tree was fitted utilizing a discrete, regional variable which defined each city as falling into one of five regions across the US (Northeast, Southeast, Midwest, Mid-Plains, West Coast). These regional variables serve as a proxy for a number of variables that may be spatially manifest—for example, differing climate across the country. Using the same number of splits (8), this tree had a similar overall fit to the a-spatial model (r^2^ = 0.3), and used similar variables (EBAS_Tourism, GP_SocialJustice, LUC_LandCons, EBAS_Institutional, GP_Economy) to differentiate grey and green cities. The geographically-stratified model did not identify the lack of trail policy initiatives, land economic barriers, and quality of life initiatives but adding an additional 3 splits (11 total) to the model reintroduces these variables, suggesting that model differences are largely due to the additional complexity introduced into the model by the geographic regions. Small differences between Mid-Plains cities and other cities emerged in terms of the relative importance of variables. Two key examples are that tourism economic base was more important in the Mid-Plains, while quality of life initiatives were more important in other cities.

## 4. Discussion and Conclusions

### 4.1. Linking Greenness to Economic Characteristics and Policy Priorities

Our results show that there are economic characteristics and policy priorities that distinguish green and grey cities. Of the general policy initiatives we tested, green cities were more likely to have social justice policy priorities, quality of life-related policy priorities, and trails initiatives; grey cities were more likely to have economic policy priorities. However, these initiatives were not enacted equally across all cities. For example, while social justice policy initiatives were important in distinguishing green from grey cities in both the ANOVA and decision tree analysis, only 11% of the cities we examined reported having them. Conversely, some policy initiatives were very common, such as economic initiatives, which were present in 73% of the cities we examined.

Both quality of life policy initiatives and trails policy initiatives were key factors in differentiating green and grey cities in our decision tree analysis though neither was highlighted as being statistically significant in the ANOVA analysis. This suggests that these types of policy priorities are helpful in distinguishing grey from green cities only in conjunction with other policy or economic conditions (in this case, cities with, and without, strong economic policy initiatives, respectively). This type of decision tree approach to policy analysis has the potential to highlight the complexity and interconnected nature of urban policy priorities and their outcomes. This may be an example of what has been termed a “causal cluster”, meaning that there are multiple corresponding forces at play in producing a particular outcome [73,74]. Further research should be done to untangle these relationships.

Both analyses agree that cities that have identified land availability as a barrier to economic growth also tend to have higher levels of vegetative greenness. A possible explanation for this may be that cities that have indicated that land is a barrier to economic growth may also have strictly enforced regulations on open space that limit land availability but increase greenness. Finally, land conservation policies were identified by both analyses as being able to distinguish green from grey cities. While this is not surprising in and of itself, the fact that other policies (*i.e.*, Open Space policies, Urban Garden policies) were not identified in either model is. One possible explanation for this disconnect is that the scope of such initiatives could be too small to be identified using the coarse spatial resolution (250 m) of our satellite-sensed vegetation data. Implementing methods for systematic, finer-scale vegetative mapping could help to overcome this challenge.

### 4.2. The Role of Spatial Variation in Vegetative Greenness

At the US scale, vegetative density showed an apparent east-west trend, following natural variability across climatologic regions. However, when spatial variability was introduced into the decision tree model, key variables—and the importance of these variables—were similar to the decision tree model which did not include spatial variability. One possible interpretation of this result is that, within cities, natural variation is less important than local irrigation efforts. Supporting this argument is recent work by Milesi [37,50], which using conservative estimates identify turf grass as the single largest irrigated crop in the United States—nearly three times larger than that of irrigated corn. Evidence can also be seen in the green lawns of desert cities such as Phoenix, and semi-arid cities such as Los Angeles.

While the distinctions between the two decision trees are small in terms of what variables are identified as being helpful in distinguishing green from grey cities, some regional variations in the importance of variables did emerge. As noted in the results, having an economic base of tourism was found to be more important (situated higher in the tree) in distinguishing green from grey cities in the Mid-Plains region than other regions. Conversely, Quality of Life initiatives and trail initiatives were more important in cities located in regions other than the Mid-Plains. While these regional differences may be indicative of how policy decisions can vary in their impact across different geographies, further work is necessary to draw causal connections. Further, explicit incorporation of climate variables across cities could better elucidate the drivers behind regional differences.

### 4.3. Decision Tree Classification

Decision tree interpretation can be challenging, and the path-dependent nature of the decision tree must be acknowledged. For example, while having conservation-based land use policies results in higher than average vegetation cover, this is only true for cities that also do not have a tourism-based economy, do not have strong economic policies, do not have trail initiatives, and do not have social justice policy initiatives. This limitation is a key reason behind the importance of splits located higher in the decision tree.

While both the ANOVA and decision tree analyses agree that having general economic policies in place is related to lower vegetative greenness, the decision tree further elucidates specific types of economic policies that may be important. For example, the decision tree suggests that having a tourism-based economy is associated with higher levels of greenness, as is an institutionally-based economy. The directionality here is not necessarily straightforward, as cities with higher levels of greenness may attract tourism and universities, while at the same time these sectors may also be more supportive of prioritizing green amenities. Further research is needed to determine how these relationships unfold in particular cities.

## 5. Conclusions

Not all urban environments are the same. Ranging from densely populated urban-industrial complexes to sparsely populated towns, the way different cities relate to their environment is reflected in their place along the continuum of urbanization. Understanding the economic and policy characteristics underlying the relationship between urbanization and vegetative land cover is one important contribution towards complicating traditionally held views of the rural-urban divide. Our results show the range of economic characteristics and policy initiatives that are associated with the urban green-to-grey continuum. These are complex relationships, as no single factor is able to completely distinguish between cities with high levels of vegetative greenness and those with low levels. Rather, a range of possible decisions and priorities face each municipality, and the confluence of these decisions can either facilitate or impede the development of a green city. While previous research has focused on understanding the differences in vegetation within cities, this paper has provided important insights into the differences in vegetation between cities along the urbanization continuum.

The aim of this paper is to complicate traditional ideas about the urban-rural divide by examining the policy priorities and economic characteristics of cities at different points along the urbanization continuum. As cities continue to grow and evolve, vegetative greenness provides a novel entry point for examining the heterogeneous nature of urbanization and the policies and priorities that guide these processes. Greenness has significant links to a variety of environmental and human outcomes and is a key feature of the new urbanization continuum. This paper identified what economic and policy differences exist between “green” and “grey” cities by employing MODIS 250 m-resolution NDVI data in conjunction with ICMA sustainability and economic survey results. We found that: (1) cities that have a high focus on economic initiatives tend to be less green; (2) cities that have a focus on social justice initiatives tend to be more green, but are very rare (11% of sample cities); and (3) the specific economic base of a city can aid in distinguishing between green and grey cities, but only under some conditions. These findings suggest that the economic characteristics and governance priorities of cities correspond on a national scale with variation in vegetative greenness, and combinations of factors—or causal clusters—underlie these relationships. Future research using finer resolution information could both validate these findings and elucidate how vegetative structure has changed over time. The new urbanization continuum is a product of economic and political decision making, and understanding these relationships is critical to realizing greener, more sustainable cities of the future.

## Figures and Tables

**Table 1:** Survey questions from the ICMA sustainability and economic surveys used to assess economic characteristics and policy priorities for each of the 373 US cities.

**Theme: General Policy Initiatives**

*Is the environment a high priority within the jurisdiction? (GP_Environment)*
*Is the economy a high priority within the jurisdiction? (GP_Economy)*
*Is social justice a high priority within the jurisdiction? (GP_SocialJustice)*
*Does the local government have an established plan for tree preservation and planting? (GP_Trees)*
*Has the community added bike and walking trails within the last 5 years?(GP_Trails)*
*Does the local government support quality of life programs to promote economic development?(GP_QualityOfLife)*

**Theme: Land Use/Cover Policies**

*Do your land use and development policies encourage mixed-use development?(LUC_Mixed)*
*Do your land use and development policies reduce fees for environmentally friendly development?(LUC_Environment)*
*Do you have an active brownfields, vacant property, or other program for revitalizing abandoned or underutilized residential, commercial, or industrial lands and buildings? (LUC_Brownfield)*
*Do you have an active land conservation program? (LUC_LandCons)*
*Do you have a program for the purchase or transfer of development rights to preserve open space? (LUC_OpenSpace)*
*Has the government taken action in regards to the use of public land for community gardens? (LUC Gardens)*

**Theme: Economic Base**

*Does Agricultural (farming and supporting industries) best describe the local government's primary economic base?(EBAS_Agriculture)*
*Does Tourism/hospitality (including travel for pleasure, business, and to visit family and friends) best describe the local government's primary economic base? (EBAS_Tourism)*
*Does Institutional (military, gov't, nonprofit, university, etc.) best describe the local government's primary economic base? (EBAS_Institutional)*
*Does technology/telecommunications best describe the local government's primary economic base? (EBAS_TechTele)*
*Does Manufacturing best describe the local government's primary economic base? (EBAS_Manufacturing)*

**Theme: Economic Barriers**

*Is the availability of land a barrier to economic development the local government has encountered? (EBAR_Land)*
*Is a lack of political support a barrier to economic development the local government has encountered? (EBAR_PoliticalSupport)*
*Is a poor quality of life (inadequate education, recreation, and arts/cultural programs) a barrier to economic development the local government has encountered? (EBAR_QualityOfLife)*

**Table 2:** Survey results, showing the average and std. deviation of responses from all cities in our sample (n = 373).

Theme: General Policy Initiatives			Theme: Economic Base		

Variable	Mean	Std. Dev.	Variable	Mean	Std. Dev.
GP_Environment	0.28	0.45	EBAS_Agriculture	0.10	0.30
GP_Economy	0.73	0.45	EBAS_Tourism	0.13	0.34
GP_SocialJustice	0.11	0.31	EBAS_Institutional	0.18	0.38
GP_Trees	0.59	0.49	EBAS_TechTele	0.09	0.29
GP_Trails	0.75	0.43	EBAS_Manufacturing	0.29	0.45
GP_QualityOfLife	0.63	0.48			
**Theme: Economic Barriers**			**Theme: Land Use/Cover**		

**Variable**	**Mean**	**Std. Dev.**	**Variable**	**Mean**	**Std. Dev.**

EBAR_Land	0.48	0.50	LUC_Mixed	0.22	0.41
EBAR_PoliticalSupport	0.11	0.31	LUC Environment	0.08	0.27
EBAR_QualityOfLife	0.05	0.21	LUC_Brownfield	0.32	0.47
			LUC_LandCons	0.32	0.47
			LUC_OpenSpace	0.21	0.41
			LUC_Gardens	0.38	0.48

**Table 3:** Results of ANOVA analysis. Significance (alpha < 0.05) indicated by a * and highlighted.

Variable	*F*	Variable	*F*
Model	5.11*	LUC_Mixed	0.80
GP_Economy	5.14*	LUC_Environment	0.32
GP_Social Justice	35.8*	LUC_Brownfield	2.11
GP_Trees	2.52	LUC_LandCons	6.01*
GP_Trails	0.00	LUC_Open Space	1.31
GP_Quality Of Life	1.15	LUC_Gardens	0.07
GP_Environment	1.70		
Variable	*F*	Variable	*F*
EBAS_Agriculture	0.25	EBAR_Land	5.61*
EBAS_Tourism	0.01	EBAR_Political Support	1.97
EBAS_Institutional	0.21	EBAR_Quality Of Life	1.60
EBAS_TechTele	1.07		
EBAS_Manufacturing	0.04		
